# Validity and reliability of the Amharic version of supportive care needs survey - short form 34 among cancer patients in Ethiopia

**DOI:** 10.1186/s12913-021-06512-2

**Published:** 2021-05-21

**Authors:** Tsion Afework, Abigiya Wondimagegnehu, Natnael Alemayehu, Eva Johanna Kantelhardt, Adamu Addissie

**Affiliations:** 1 School of Public Health, Department of Preventive Medicine, Addis Ababa University, Addis Ababa, Ethiopia; 2 Faculty of Medicine, Palliative Care Unit, Hawassa University, Hawassa, Ethiopia; 3 Institute of Medical Epidemiology, Biostatistics and Informatics, Martin-Luther-University, Halle (Saale), Germany; 4 Department of Gynecology, Martin-Luther-University, Halle (Saale), Germany

**Keywords:** Convergent validity, Confirmatory factor analysis, Discriminant validity, Ethiopia, Supportive care needs, SCNS-34

## Abstract

**Objectives:**

Supportive care needs survey short form has a total of 34 items that have 5 domains that measure the unmet needs of cancer patients. It is important to validate this tool since there are differences in culture, geographic areas, and clinical care service which influence patients’ needs. Therefore, this study aimed to assess the construct validity and reliability of the tool.

**Methods:**

The study was conducted among 170 cancer patients from April 1st to 30th 2019 in Hawassa hospital, South Ethiopia. Confirmatory factor analysis was done using fit indices. Convergent and discriminant validity was evaluated using average variance extracted and maximum shared variance respectively. Known group validity was checked using the Mann-Whitney U test. The reliability of the instrument was examined using Cronbach’s alpha.

**Results:**

Domains except for health system and information, and patient care and support maintained convergent and divergent validity. The remaining validity was maintained after removing items that were redundant and double loading. The average variance extracted of domains varied from 0.52–0.81. The Square of correlation between constructs was lower than the average variance extracted for the constructs. The tool had reliability *r* = 0.932. The root mean square error of approximation was 0.057, comparative fit index 0.954, and the other fit indices were also indicating a good fit. Known groups difference was seen by age and type of treatment taken across the different domains.

**Conclusion:**

After the health system and information, and patient care, and support domain validity issues were corrected by removing 8 items, the reduced tool was found to be a valid and reliable tool. The validated tool will be valuable if included in routine cancer care in our clinical settings.

**Supplementary Information:**

The online version contains supplementary material available at 10.1186/s12913-021-06512-2.

## Introduction

The burden of cancer continues to rise because of the increase in life expectancy and world population alongside the adoption of cancer-causing behaviors [1]. According to the global organization board of cancer association network (GLOBOCAN) in 2018, the global cancer burden has risen to 18.1 million cases [2]. In Africa, the World Health Organization (WHO) projection suggests that cancer incidence will double to 1.28 million new cases per year by 2030 [3]. The incidence of cancer in Ethiopia in 2018 was estimated at around 67,573 cases [4].

Supportive care is a service that is provided at any point along the continuum of care for cancer patients to meet their needs in different aspects like physical, emotional, social, informational, or spiritual needs [5]. Supportive care needs survey (SCNS) measures the perceived needs of adult cancer patients across different needs [6, 7].

The psychological domain assesses needs related to support in coping with cancer and emotions, the health system and information domain assess needs related to information about the disease, diagnosis, treatment, and follow-up. Dealing with physical symptoms, side effects of treatment, and performing usual tasks and activities is assessed by the physical and daily living domain. The patient care and support domain evaluate patients’ needs for health professionals to show compassion to their physical and emotional needs, privacy, and choice and, the sexuality domain measures needs of cancer patients related to change in sexual feelings and sexual relationships [6].

In different studies magnitude of unmet needs varied from 27 to 65% [8–10]. Psychological [11–13] and/or the health system and information domains [14, 15] were the domains with the most frequently reported unmet needs. The theoretical model of this tool was on a priori estimates of scale constructs which are based on the Cancer Needs Questionnaire constructs [16].

The SCNS was first developed in the late 1990s and consists of a 59-item long-form, a 31-item short-form survey, and the recently developed 34-item short-form survey (SCNS-SF34) [6]. It was developed in Australia [6, 17, 18] and was translated and validated in different languages including German [19], French [20], Japanese [21], and Chinese [22], showing good internal validity and reliability but different factor structure [22], proving the existence of cultural differences in scale understanding. Supportive care needs (SCNs) among cancer patients can be influenced by cultural variations and the quality of care they receive in different facilities [23]. A study done on unmet supportive care needs: a cross-cultural comparison between Hong Kong Chinese and German Caucasian women with breast cancer, concluded that culture-specific differences in supportive care needs exist. This indicated that planning for cancer supportive care services or interventions to reduce unmet needs must consider cultural and/or health service contexts [23]. Also, another study argued that quality of life in cancer patients from different cultures offers the potential for fascinating insights that will guide the development of culturally appropriate supportive care to improve patient well-being, this will help to explain which impacts of cancer and cancer treatments aspects are culture-specific [24].

There was a review of the needs assessment literature in the oncology field, and it was found that no instrument met all of the following criteria for an acceptable needs assessment tool, then Cancer Needs Questioner was developed, then SCNS was developed, SCNS was able to measure the multidimensional and comprehensive range of needs, directly assesses patients’ perceptions of their needs, assesses whether issues of need have been experienced, which issues remain unmet needs and the magnitude of such needs on one response scale, measures outcomes within a defined temporal context, needs assessment enables the identification of patient subgroups with higher-level needs, thereby potentially enabling prevention or at least reduction of problems through appropriate early intervention [25, 26]. SCNS also demonstrates acceptable reliability and validity and is user-friendly.

Information about SCNs of cancer patients comes primarily from developed countries [19, 20]. whereas, in developing countries, this type of information is limited. This is partially explained by the fact that there are no rigorously validated instruments to measure patients’ needs [27]. To accurately identify the needs of cancer patients, SCNs instruments with proven psychometric properties are essential. To our knowledge, a specific tool that assesses the supportive care needs of cancer patients has not been validated in an Ethiopian language. Therefore, this study aimed to assess the construct validity and reliability of the SCNS-SF34 among the Ethiopian Amharic-speaking population.

## Methods

### Study design, area, and period

A cross-sectional study design was employed at Hawassa comprehensive specialized hospital. It is one of the teaching and referral hospitals providing cancer treatment for the newly established Sidama region, southern nation’s nationalities, and peoples of Ethiopia and neighboring areas of Oromia. The hospital was established in 1998 GC and accommodates about 400 beds for inpatient service of which 316 are functional. The oncology unit and palliative care units are two of the recently established service areas in the hospital. Before 2019 the palliative care service was given under the oncology unit but currently, the palliative care unit was established for the first time in the region. In the oncology unit, there is a provision of only medical oncology services. Yearly more than 700 new patients visited the center. Both the oncology and palliative care units are the only service providers in the southern part of the country. The study was conducted, from April 1st to 30th 2019.

### Participants

The inclusion criteria were; cancer patients with pathologically confirmed malignancy, being treated outpatient or inpatient, either on chemotherapy, hormonal therapy, surgery, or radiotherapy and able to speak and listen Amharic, diagnosed at least 3 months ago and who was 18 years old and above were included. Patients who had acute pain and with known psychiatric illnesses were excluded from the study.

### Instrument

Based on the recommended guideline for tool translation [28], the SCNS-SF34 was translated by a certified legal translator native speaker of Amharic and was back-translated into English by another legally certified translator. Then two independent reviewers checked for inconstancies. The first one was a PhD candidate and the second person was a master’s student. Both had health backgrounds and can adjust terms from the perspective of clinical practice to ensure medical equivalence with the original scale. Both health professionals conducted a comparative analysis of the translated and back-translated versions. A consensus meeting between the translation team resolved any inconsistencies. Finally, it was given back to the legal translation team, then the tool was compared with the original scale, and was modified repeatedly until the English version was as similar as possible to the original scale. we have done a pilot test of the tool among 10 cancer patients, then after collecting feedback from data collectors which they received from the patients about the items wording and their understanding, some of the items were again modified.

### Sample size determination and sampling procedure

The sample size was determined using the assumption of 5–10 participants /item for checking factor structure and validity of items. Since SCNS-SF34 had 34-items, this study was conducted among 170 participants [29]. Cancer patients that came to the oncology unit were consecutively included until the required sample size was achieved.

### Data collection procedure

Physical and daily living needs have 5 items, psychological-needs 10 items, health system and information-needs 11 items, patient care and support-needs 5 items and sexuality-needs have 3 items. Participants were asked to indicate the level of their need for help over the last month concerning “having cancer” based on a 5-point Likert scale (1 = not applicable or no need, 2 = no need or satisfied, 3 = low need, 4 = moderate need, and 5 = high need).

Socio-demographic and clinical characteristics of patients were collected using an interviewer-administered structured questionnaire and from the patient’s chart. The data was collected by experienced clinical BSc nurses. They were trained for 2 days based on a manual prepared by the principal investigator. All collected data were examined for completeness and consistency by the immediate supervisor at the hospital.

### Data management and analysis

Data was entered into EPI INFO version 4.4.3.1 software packages then exported to SPSS version 25 for analysis. The descriptive part was summarized using frequency distribution and proportions for categorical variables; other continuous variables were described using means, standard deviations, median and interquartile range based on the assumption of normality. Wealth index was computed from 19 questions which included questioners related to housing conditions & household characteristics, principal component analysis (PCA) was used to reduce the number of variables measuring wealth, finally, PCA had divided it equally to five quintiles each had a share of 20%.

First, the adequacy of the sample was estimated by Kaiser-Meyer-Olkin (KMO) test. KMO indicated the adequacy of the sample for factor analysis and was considered adequate if above 0.5. The construct validity was evaluated by subjecting the items of the instrument to confirmatory factor analysis (CFA) using SPSS Analysis of Moment Structures (AMOS) version 23 software. The CFA was done to confirm whether the items are a good measure of the latent constructs and to check whether the items loaded on the proposed constructs and to see for appropriateness of the factor structure [30]. The fit indices used for CFA were relative chi-square < =2, the standardized root means square residual with a cut of the value of < 0.06, comparative fit index > = 0.90 roots mean square error of approximation *<*.05 and *<* .08 [30–32].

Microsoft Excel was also used to calculate the convergent and divergent validity. Convergent validity in this study aimed to see if items of the same construct either converge and share high proportion variance with other items of different construct but in the same measurement. Neither one measures the amount of variance that is captured by the construct with the amount of variance due to measurement error. The average variance extracted (AVE) provides this information. The formula for AVE is the sum of the square of factor ladings divided by the number of items. It is calculated from CFA output using standardized regression weights. Each construct was evaluated against its correlation with other constructs, each factor AVE should be greater than 0.5 to indicate good convergent validity. An item is considered for deletion if factor loadings were between 0.40 and 0.70 and if it contributed to an increase in composite reliability (CR) and AVE. [33, 34] Discriminant validity was established where maximum shared variance (MSV) which is the square of the correlation between constructs was lower than AVE for all the constructs or square root of the AVE for each of the latent variables should be higher than the highest correlation with any other latent variables, if that is the case, discriminant validity is established at the construct level. This rule is known as Fornell–Larcker criterion [34]. The internal consistency of the instrument was examined based on Cronbach’s alpha, the general rule of thumb is that a Cronbach’s alpha of .70 and above is good, .80 and above is better, and .90 and above is best [35]. Known group comparisons were carried out using the Mann-Whitney U test since the domain scores violated normality assumptions. We first compared the SCNS-SF 26 domain scores between participants older and younger age group (18–64 vs ≥ 65). We then compared the SCNS-SF34 scores by gender, comorbidity status, stage of cancer, and type of treatment the patient is currently on. It was hypothesized that younger patients and females would have higher scores in unmet needs and from the clinical characteristics those with comorbidity, on chemotherapy and those who have advanced cancer would report higher levels of unmet needs than their counterparts [27, 36, 37]. If m equals the number of questions in a scale and k is the value of the maximum response for each item, the standardized score for each domain is obtained by summing the individual items, subtracting m, and then multiplying the resulting value by 100/(m × (k-1)) [6]. A *p*-value of < 0.05 was used to declare a statistically significant.

## Results

### Socio-demographics of the respondent

Three fourth of participants were females (72%), the median age was 40, and IQR (32–50). Two-third (64%) of the patients came from urban residences. One-fourth (25.3%) of the participants had no formal education (Table 1).

**Table 1:** Socio-demographic of the respondents in Hawassa comprehensive specialized Hospital, SNNPR, Ethiopia, 2019

Variables	*n* = 170	Percent (%)
Age (Median, IQR ^a^)	40 ± (32–50)	
Sex		
Female	122	71.8
Male	48	28.2
Residence		
Urban	108	63.5
Rural	62	36.5
Region		
Oromiya	52	30.6
Amhara	3	1.8
SNNPR ^b^	115	67.6
Marital status		
Married	132	77.6
Never married	14	8.2
Divorced/separated	9	5.3
Widowed	15	8.8
Education		
No education	43	25.3
Can read and write	15	8.8
Primary	41	24.1
Diploma and degree	45	26.5
Secondary	25	14.7
Masters and above	1	0.6
Occupation		
Housewife	55	32.4
Governmental employee	44	25.9
Farmer	32	18.8
Merchant	14	8.2
Student	9	5.3
Others ^c^	16	9.4
Wealth index		
Lowest	32	20
Second	32	20
Middle	32	20
Fourth	32	20
Highest	32	20

^a^ Interquartile range, ^b^ Southern Nations, Nationalities, and People’s Region, ^c^ non -governmental organization, un-employed, daily laborer

### Clinical characteristics of the respondent

Half (49%) of the patients were diagnosed with breast cancer followed by gastro-intestinal cancers 39 (22.9%) and hematologic cancers 17 (10%) (See Additional file 1).

### Factor ladings

Higher factor loadings were seen in all domains (> 0.5) (See Additional file 2).

### Confirmatory factor analysis

KMO of the tool was 0.89, which shows the data was suited for factor analysis. Factor structure and model fit of the data was checked on AMOS, all the fit indices were below their cut of value (CFI = 0.789), and (RMSEA = 0.103), the raw *χ*^2^ was 1451.353 and *df* was 517 with *p*-value < 0.0001) indicating that the data poorly fit (Table 2).

**Table 2:** Confirmatory factor analysis in Hawassa comprehensive Specialized Hospital, Hawassa, Ethiopia, 2019

	X^**2**^	***p-*** value	Df ^a^	REMSEA^b^	CFI ^c^	SRMR^d^	CMIN/DF^**e**^
**Baseline model**	1451	0.0001	517	0.103	0.789	0.102	2.807
**Model 1**^**f**^	817.7	0.006	496	0.062	0.927	0.098	1.649
**Model 2**^**g**^	429.9	0.123	276	0.057	0.954	0.092	1.558

^a^ Degree of freedom, ^b^ Root mean square error of approximation, ^c^ Comparative fit index, ^d^ Standardized root mean square residual, ^e^ relative chi-square, ^f^ model 1: after correlation of residuals modified SCNS 34 model fit, ^g^ model 2: the modified and final model fit after items removed and 26 items remained

Nerveless, there was room for improvement by modification indices, as suggested by AMOS correlating residuals [38]. After correlating each residual, the model was re-run again each time.

**Fig. 1:**
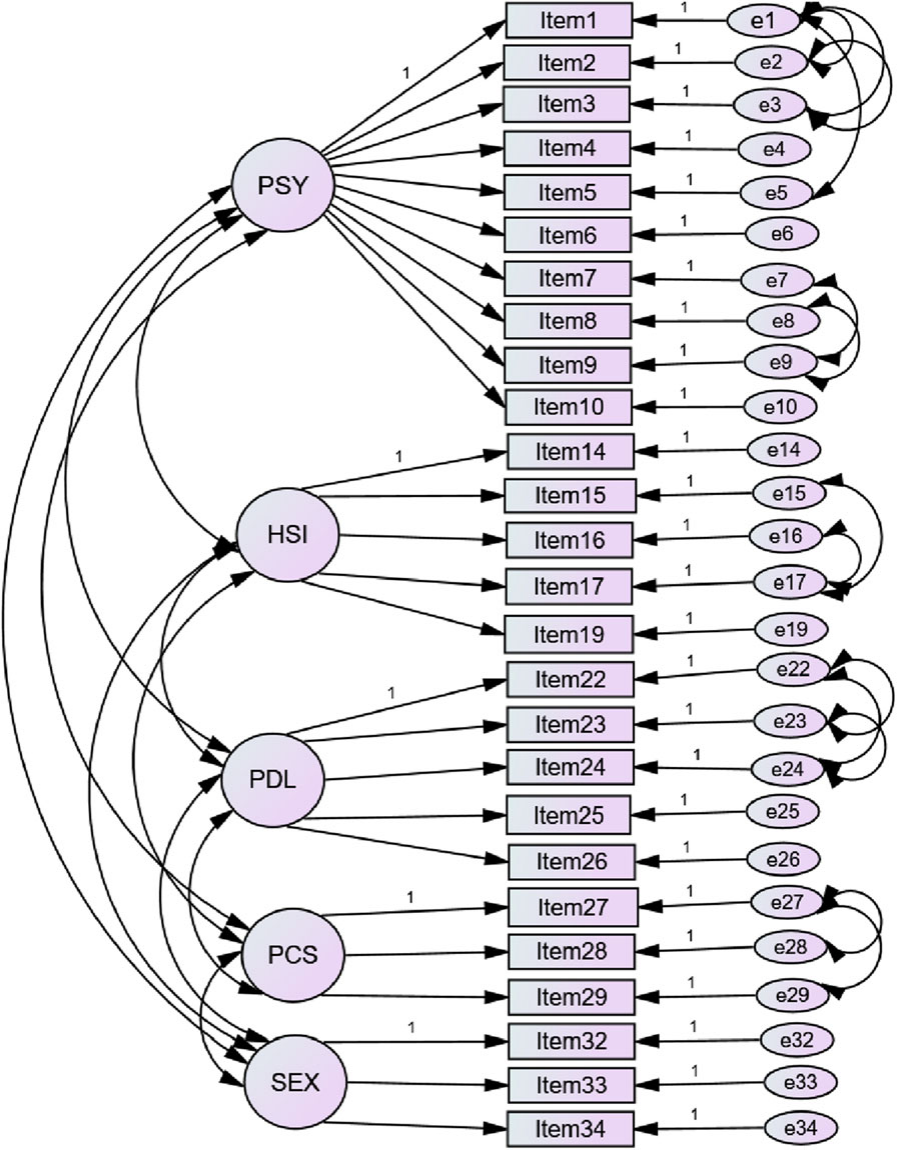
Model fit after residual correlations tool in Hawassa comprehensive Specialized Hospital, Addis Ababa, Ethiopia, 2019

Totally 21 residuals were correlated to reach fairly acceptable fit indices, (CFI = 0.927), CMIN/DF = 1.649), and (RMSEA = 0.062), the raw *χ*^2^ was 817.7 and *df* was 496 with *p*-value < 0.006) (Table 2). The higher correlation was found between error term 11 and 12 (0.74), between error term 2 and 3 (0.53), between error term 16 and 17 (0.53), between error term 22 and 24 (0.52), and between error term 1 and 3 (0.51) (Fig. 1).

### Correlation between latent factors

The correlation between domains ranged from 0.1–0.81. Health systems and information and patient care and support had a higher correlation between them compared to the other factors that were 0.81. The lowest correlation was between physical and daily living and sexuality domains which were 0.1 (See Additional file 3).

### Convergent validity

Psychological, physical and daily living, and sexuality domain had good convergent validity with an estimated AVE value of PSY (0.61), PDI (0.59), and SEX (0.81). While the health system and information domain had poor convergent validity with AVE 0.45. Therefore, those items with low factor loading were removed turn by turn to improve the AVE value. Item 20 was first removed since it had the lowest factor loading (0.54) and AVE slightly improved to 0.46. Then, item 11 (factor loading 0.58) was removed and AVE became 0.48. But still, AVE was low so, we removed items 13 and 12 which had 0.60 and 0.61 factor loading respectively. Finally, convergent validity was maintained for this domain at AVE =0.51. Similarly, the patient care and support domain had an initial AVE value of 0.46. However, this value changed to 0.51 after the removal of item 30 (Table 3).

**Table 3:** Convergent and divergent validity of SCNS-26 tool in Hawassa comprehensive Specialized Hospital, Hawassa, Ethiopia, 2019

	AVE	MSV	PSY	HSI	PDI	PCS	SEX
**PSY** ^a^	0.610	0.358	**0.781**				
**HSI** ^b^	0.517	0.492	0.598	**0.719**			
**PDL** ^c^	0.585	0.126	0.355	0.263	**0.765**		
**PCS** ^d^	0.517	0.492	0.511	0.701	0.307	**0.719**	
**SEX** ^e^	0.806	0.092	0.303	0.151	0.059	0.134	**0.898**

^a^ Psychological, ^b^ Health information, ^c^ physical and daily living, ^d^ patient care, and support, ^e^ Sexuality

### Divergent validity

Except for health system and information and patient care and support, other domains had good divergent validity. Psychological domain had MSV = (0.09, 0.26, 0.13, and 0.36) physical and daily living MSV = (0.003, 0.09, 0.13 and 0.07) and sexuality domain MSV = (0.02, 0.003, 0.02 and 0.09). The square root of AVE for these domains was more than the correlation of the latent variables; psychological (0.78), physical and daily living (0.76), and sexuality domain (0.898), Therefore, the divergent validity of these three domains was confirmed by both methods.

The divergent validity for the health system and information domain was assessed. MSV (square of the latent variable correlation between HSI and PCS) was (0.64), which was greater than the AVE value of 0.51 and 0.5 of HSI and PCS respectively, Since AVE < MSV divergent validity problem has occurred. The square root of AVE for both domains was 0.71, which was less than the correlation of the latent variables between them (0.80), in that case since the square of AVE is less than the correlation of the latent variables, it had shown there is a divergent validity issue in both domains.

The items were checked for double loading as a result items 18, 21 from the HSI domain, and 31 from the PCS domain were found to have double loading. Each item was removed turn by turn until the desired result is achieved. When item 21 was removed AVE of health system information improved to 0.523, MSV decrease to 0.60, square of AVE increase to 0.72, but still (AVE < MSV), when item 31 was removed, AVE of patient care and support was improved to 0.527, MSV decrease to 0.54, and the square root of AVE also increased to 0.76 which was higher than the correlation between constructs of patient care and support and health system and information (0.73), finally when item 18 was removed the MSV decrease to (0.49), square of AVE for the health system and information was also higher (0.72) than the correlation between latent variables of the health system and patient care and support domain, which was 0.7. After the above steps, divergent validity of both the health system and information and patient care and support domain was established (Table 3).

After convergent and divergent validity was maintained the final SCNS-26 item was subjected to confirmatory factor analysis, and the model fit indices even got better. All fit indices have been fulfilled showing that the data fits the model (Table 2).

The final SCNS-26 items were, the original 10 items from the psychological domain, from the health system information domain 5 items were obtained, the original 5 items were achieved from the third domain physical and daily domain, the fourth dimension, patient care, and support obtained only 3 items. The final domain obtained was the sexuality domain, which was similar to the original one. The final maintained dimensions will give a clinical view about needs of cancer patients related to emotions and coping with the disease, measures the need for information about disease, medication benefit and side effect, the result of the investigation, also explore needs related to coping with physical symptoms, side effects of treatment.

### Reliability of the scale

The internal consistency of the new SCNS-26 was checked using Cronbach’s alpha, all the domains of SCNS had a value above 0.7 overall the tool had good reliability (0.932) (See Additional file 4).

### Known group difference in the SCNS-26 domains

Five known-group comparisons were carried out using the Mann-Whitney U test. Younger patients have higher unmet needs in the sexuality domain compared to older ones. There was a difference in mean rank score among patients on chemo/radiotherapy in all domains except in physical and daily living needs compared to those who were on hormonal or surgical treatment (Table 4).

**Table 4:** Known group validity of SCNS-26 tool in Hawassa comprehensive Specialized Hospital, Hawassa, Ethiopia, 2019

Variable	Physiological needs		Health system information needs		Physical & daily living needs		Patient care & support needs		Sexuality needs	
	Mean rank	***P***-value	Mean rank	***P***-value	Mean rank	***P***-value	Mean rank	***P***-value	Mean rank	***P***-value
Sociodemographic characteristics										
Age		0.517		0.557		0.441		0.232		**0.022 Ҩ**
18–64	86.31		86.24		86.46		87.00		87.90	
≥ 65	78.18		78.88		76.85		72.03		63.91	
Sex		0.899		0.498		0.958		0.291		0.692
Male	86.26		81.45		85.19		79.18		83.52	
Female	85.20		87.09		85.62		87.99		86.28	
Clinical characteristics										
Comorbidity		0.380		0.370		0.976		0.877		0.911
Yes	78.76		78.65		85.27		84.32		86.21	
No	87.12		87.15		85.55		85.78		85.33	
Type of treatment		**0.017 Ҩ**		**0.003 Ҩ**		0.302		**0.0001 Ҩ**		**0.009 Ҩ**
Chemotherapy/radiotherapy	90.55		91.71		87.68		94.27		90.12	
Hormonal/surgery	69.60		65.95		78.65		57.90		70.95	
Stage of cancer		0.386		0.268		0.537		0.759		0.194
Early stage (I-II)	90.04		91.28		82.28		87.10		79.84	
Late stage (III-IV)	83.15		82.51		87.17		84.67		88.43	

Ҩ statistically significant at *p*-value < 0.05

## Discussion

This study aimed to assess the construct validity and reliability of the SCNS-SF34 among the Ethiopian Amharic-speaking population.

During the development of the original SCNS-SF34 in Australia, the authors performed principal factor analysis and 72.1% of the total variance was identified [6]. Similarly, in our study, the five-factor SCNS-SF34 explained 66.7% of the variance. In a Turkish study, it was seen that the total variance explained from 29 items with four factors was 68.83% [39]. Variability was better explained in the Turkish study since it was only 29 items, the other reason might be the difference in culture, age, and gender, cancer type, which may strongly influence how people experience supportive care needs [37].

In a study done in France on 384 breast cancer patients, indicators of fit from confirmatory factor analysis on the SCNS-SF34- five-factor model were fairly acceptable [x^2^(517) = 1616.7, *p-value* 0.001; RMSEA = 0.076; CFI = 0.96;] [20]. But in our study, the model fit the data poorly. In the French study after correlation with residuals, the model provided an improvement to a more acceptable fit: (x^2^: (505) = 1023.9, *P <* 0.001; RMSEA = 0.052; CFI = 0.98 [20]. In our study, a total of 21 residuals were correlated to reach fairly acceptable fit indices. These results led to a convulsion that residuals were correlated indicating redundancy among items, which was supported by finding from other factor analysis studies on SCNs [20, 37].

Bredart A et al., found that correlations between SCNS-SF34 factors ranged from 0.26 to 0.86, with high correlations between the ‘health system and information, and ‘patient care and support’ (*r* = 0.86) than between the ‘psychological’ and ‘physical and daily living’ factors (*r* = 0.77) [20]. Similarly, in our study higher correlations were seen between ‘health system and information, and ‘patient care and support factors.

This might be reasoned as these two domains probably were understood almost similarly by the respondents and some items were redundant. Supporting this, a study in China found a four factor model after these two domains loaded together [22].

In a study done in Japan, two items: ‘Hospital staff attending promptly to your physical needs’ and ‘Hospital staff acknowledging, and showing sensitivity to, your feelings and emotional needs,’ originally in the patient care and support needs factor, loaded evenly on both patent care and support and health system information factor [21]. In another validation study in Germany, it was found that item 30 which is “Hospital staff attending promptly to your physical needs” had stronger cross loadings on the psychological domain [22]. This was seen in our result too, the convergent validity of patient care and support domain was maintained as item 30 was removed as a result of its lower factor loading as this item was not correlating with the other items of the same domain.

Item 20 “To be treated in a hospital or clinic that is as physically pleasant”, had also lower factor lading and was eventually removed, this might be due to, our study setting was the only governmental hospital giving oncology service for that area, so patients did not perceive being in a pleasant hospital as there most important need rather getting the chance to be treated. Item 11 “To be given written information about the important aspects of your care”, item 12 “To be given information (written, diagrams, drawings) about aspects of managing your illness and side-effects at home”, and item 13 “To be given explanations of those tests for which you would like explanations “were all the items that affected the convergent validity of health system and information domain, this might be as a result of lower educational status of our participants leading to less perceived unmet needs towards this items.

In our study, there was a problem of divergent validity on the health system and information and patient care and support domains. This was supported by other studies, as items found under one domain were strongly correlating with the other domain items [22, 39]. One Mexican study found a similar thing with ours, that item 31 was correlating with other construct and double loaded so this item was removed then divergent validity was preserved [27].

A study done by Schofield et al. in Australia, on prostate cancer patients, reported that items 18 and 19 originally allocated to the patient care and support domain did not load (loading <.30) to one of the domains at all [40]. This shows that these items were not measuring what they are supposed to measure in their domain. Contradicting to this, in our study we did not have items that did not load at all, the reason might be the cut-off value Schofield et al. used were lower which was a bit conservative as compared to our study which was taken as loading < .4. The other reason might be the former study included only prostate cancer patients in contrast to our study which included all cancers.

Exploratory factor analyses on SCNS-SF34 showed that the four-factor structure, with domains on health systems and information and patient care and support, were combined in one domain and were the best model for use in head and neck cancer patients [22]. Other validation studies proposed the (slightly adjusted) five-factor structure of the original factor structure model. Although these studies also acknowledged some difficulties or inconsistencies when replicating the five-factor structure [21, 22, 27, 40]. It can be assumed that one universal factor structure for the SCNS-SF34 is unlikely [41]. Besides, based on cultural differences, addition, removal, or alteration of items can occur during validation of a tool [42].

A study conducted in Australia in 2012, showed SCNS-SF34 has several items within factors that is redundant suggesting that by removing these items, a questionnaire with a more balanced item content within factors could be created [43].

The internal consistency of the constructs in our study ranged from good- strong, which exceeded the coefficient criteria set at 0.7. This finding was similar to the original validation study done in Australia and Turk [6, 39].

### Strength and limitation

Our finding should be interpreted with some limitation since in Hawassa hospital there is no radiotherapy service, and the unmet supportive care needs of patients that are on radiotherapy treatment were not addressed. Due to the limited time and budget, test-retest reliability, content validity of this study was not assessed and the sample size for the study was determined using the assumption of 5 participants /item, this might be one of the reasons that we have not achieved satisfactory model in the first few attempts.

To our knowledge, this is the first SCNS-SF34 tool validation study in Ethiopia, possibly in Africa. The fact that the study incorporates a heterogeneous sample of patients in terms of sex, education, primary cancer site, cancer diagnosis duration, stage, and treatment, and having a complete response rate can contribute to results validity. In addition to that, our study was done at the first regional referral hospital for cancer treatment in Ethiopia where patients attending belong to a large variety of ethnic groups. This assures the representativeness of our findings.

## Conclusion

In conclusion, the SCNS-SF34 revealed both convergent and divergent validity issues raised from health system information and patient care and support domains. This study found - after removal of items 20, 11,13,12,18, and 21 from the HSI domain and items 30 and 31 from the PCS domain – that the validity for both domains was assured together with all the other three domains. The tool was also reliable. Therefore; The reduced SCNS-SF34 with a five-factor structure was found to be a valid and reliable tool to measure the supportive care needs of cancer patients in Ethiopia, and also it is suggested that other studies expand the sample, apply repeated measurements, check for the content validity, construct validity of SCNS with to other scales, use multi-step method approach for cross-cultural translation and further verify and develop the Amharic version of the SCNS-34 tool. The SCNS-SF-34 tool ought to be validated by other languages spoken by Ethiopian and with its limitations, this is the first Amharic version SCNS-34 tool and we believe the tool will be valuable if included in routine cancer care in our clinical settings. Information about the magnitude of unmet needs can inform health service planners to redesign or guide the development of new services and guidance for new strategies for health facilities to address problems that arise because of cancer. Cancer not only affects patients but also their caregivers, encompassing partners, family members, and close friends. The SCNS-34 should be embedded to clinical routine, to provide integrated comprehensive supportive care management to patients, as the tool amplify where actions and resource allocation are necessary for healthcare settings to help the patients overcome their difficulties and could potentially reduce the burden of this disease in the long run and thereby improve patients and caregivers quality of life [16, 44].

## Supplementary Information

**Additional file 1: Table 1.** Clinical characteristics of the respondents in Hawassa comprehensive specialized Hospital, SNNPR, Ethiopia, 2019. This table describes cancer and other clinical characteristics of the cancer patients.

**Additional file 2: Table 2.** Standardized Regression Weight for SCNS Tool in Hawassa comprehensive specialized Hospital, SNNPR, Ethiopia, 2019. This is output from AMOS; it shows how each of the observed variables loads on each construct. Each load more than 0.5.

**Additional file 3: Table 3.** Correlation between latent factors in Hawassa comprehensive Specialized Hospital, Hawwasa, Ethiopia, 2019. This shows if whether the five latent factors correlate with each other or not, which shows there is a high similarity in the items if there is a higher correlation.

**Additional file 4: Table 4.** Reliability of the scale of SCNS-26 tool in Hawassa comprehensive Specialized Hospital, Hawassa, Ethiopia, 2019. Reliability was checked for only the remaining 26 items, which the result shows, all the construct had good reliability, which yields the same result on repeated measurements.

## Data Availability

The datasets used and/or analyzed during the current study are available from the corresponding author on reasonable request.

## Abbreviations

GLOBOCAN: Global organization board of cancer association network
WHO: World Health Organization
SCNS-SF34: Supportive care needs survey short form 34
KMO: Kaiser-Meyer-Olkin
CFA: Confirmatory factor analysis
AMOS: Analysis of Moment Structures
AVE: Average variance extracted
CR: Composite reliability
MSV: Maximum Shared Variance
DF: Degree of freedom,
REMSEA: Root mean square error of approximation
CFI: Comparative fit index
SRMR: Standardized root mean square residual
CMIN/DF: Relative chi-square
PSY: Psychological
HSI: Health system information
PDL: Physical and daily living
PCS: Patient care and support
SEX: Sexuality
