# Supplementary material for: Validity and reliability of the Amharic version of supportive care needs survey - short form 34 among cancer patients in Ethiopia
Source: BMC Health Serv Res. 2021 May 21;21:484. doi: 10.1186/s12913-021-06512-2 (PMC8138921; doi:10.1186/s12913-021-06512-2)
Supplement: Supplementary file 1 — Additional file 1: Table 1. Clinical characteristics of the respondents in Hawassa comprehensive specialized Hospital, SNNPR, Ethiopia, 2019. This table describes cancer and other clinical characteristics of the cancer patients. [file 12913_2021_6512_MOESM1_ESM.docx]

# Validity and reliability of the Amharic version of supportive care needs survey - short form 34 among cancer patients in Ethiopia

Tsion Afework^*^, Abigiya Wondimagegnehu , Natnael Alemayehu , Eva Johanna Kantelhardt^,^ Adamu Addissie

Table 1: Clinical characteristics of the respondents in Hawassa comprehensive specialized Hospital, SNNPR, Ethiopia, 2019

| Variables | n=170 | P(%) |
| --- | --- | --- |
| Type of cancer |  |  |
| Breast | 84 | 49.4 |
| Gastro-intestinal | 39 | 22.9 |
| Gynecologic | 14 | 8.2 |
| Hematologic | 17 | 10.0 |
| Others ^d^ | 16 | 9.4 |
| Type of treatment (current) |  |  |
| Chemotherapy | 128 | 75.3 |
| Hormonal therapy | 30 | 17.6 |
| Surgery | 11 | 6.5 |
| Radiotherapy | 1 | 0.6 |
| Stage of cancer |  |  |
| Stage one | 15 | 8.8 |
| Stage two | 43 | 25.3 |
| Stage three | 57 | 33.5 |
| Stage four | 55 | 32.4 |
| History of metastasis |  |  |
| No | 108 | 63.5 |
| Yes | 62 | 36.5 |
| History of recurrence |  |  |
| No | 152 | 89.4 |
| Yes | 18 | 10.6 |
| History of co-morbidity |  |  |
| No | 137 | 80.6 |
| Yes | 33 | 19.4 |

^d^ Head and neck, Genitourinary, Skin, Endocrine, Respiratory
