# Supplementary material for: Validity and reliability of the Amharic version of supportive care needs survey - short form 34 among cancer patients in Ethiopia
Source: BMC Health Serv Res. 2021 May 21;21:484. doi: 10.1186/s12913-021-06512-2 (PMC8138921; doi:10.1186/s12913-021-06512-2)
Supplement: Supplementary file 2 — Additional file 2: Table 2. Standardized Regression Weight for SCNS Tool in Hawassa comprehensive specialized Hospital, SNNPR, Ethiopia, 2019. This is output from AMOS; it shows how each of the observed variables loads on each construct. Each load more than 0.5. [file 12913_2021_6512_MOESM2_ESM.docx]

# Validity and reliability of the Amharic version of supportive care needs survey - short form 34 among cancer patients in Ethiopia

Tsion Afework^*^, Abigiya Wondimagegnehu, Natnael Alemayehu, Eva Johanna Kantelhardt^,^ Adamu Addissie

Table 2: Standardized Regression Weight for SCNS Tool in Hawassa comprehensive specialized Hospital, SNNPR, Ethiopia, 2019

| **Domain** | **Item** | **Name of the item** | **Estimate** |
| --- | --- | --- | --- |
| **Psychological needs** | Item 1 | Anxiety | .782 |
|  | Item 2 | Feeling down or depressed | .787 |
|  | Item 3 | Feelings of sadness | .792 |
|  | Item 4 | Fears about the cancer spreading | .837 |
|  | Item 5 | Worry that the results of treatment are beyond your control | .749 |
|  | Item 6 | Uncertainty about the future | .862 |
|  | Item 7 | Learning to feel in control of your situation | .840 |
|  | Item 8 | Keeping a positive outlook | .813 |
|  | Item 9 | Feelings about death and dying | .781 |
|  | Item 10 | Concerns about the worries of those close to you | .661 |
| **Health system**  **and information needs** | Item 11 | To be given written information about the important aspects of your care | .630 |
|  | Item 12 | To be given information (written, diagrams, drawings) about aspects of managing your illness and side-effects at home | .658 |
|  | Item 13 | To be given explanations of those tests for which you would like explanations | .561 |
|  | Item 14 | To be adequately informed about the benefits and side-effects of treatments before you choose to have them | .727 |
|  | Item 15 | To be informed about your test results as soon as feasible | .665 |
|  | Item 16 | To be informed about cancer which is under control or diminishing | .737 |
|  | Item 17 | To be informed about things you can do to help yourself get well | .813 |
|  | Item 18 | To have access to professional counseling (eg, psychologist, social worker, counselor, nurse specialist) if you/family/friends need it | .750 |
|  | Item 19 | To be treated like a person, not just another case | .635 |
|  | Item 20 | To be treated in a hospital or clinic that is as physically pleasant as possible | .598 |
|  | Item 21 | To have one member of hospital staff with whom you can talk to about all aspects of your condition, treatment, and follow-up | .683 |
| **Physical and daily living needs** | Item 22 | Pain | .677 |
|  | Item 23 | Lack of energy/tiredness | .479 |
|  | Item 24 | Feeling unwell | .675 |
|  | Item 25 | Workaround the home | .969 |
|  | Item 26 | Not being able to do the things you used to do | .957 |
| **Patient care and support needs** | Item 27 | More choice about which cancer specialist you see | .640 |
|  | Item 28 | More choice about which hospital you attend | .754 |
|  | Item 29 | Reassurance by medical staff that the way you feel is normal | .716 |
|  | Item 30 | Hospital staff to attend promptly to your physical needs | .559 |
|  | Item 31 | Hospital staff to acknowledge, and show sensitivity to, your feelings and emotional needs | .778 |
| **Sexuality needs** | Item 32 | Changes in sexual feelings | .956 |
|  | Item 33 | Changes in sexual relationships | .974 |
|  | Item 34 | To be given information about sexual relationships | .745 |
