# Supplementary material for: Validity and reliability of the Amharic version of supportive care needs survey - short form 34 among cancer patients in Ethiopia
Source: BMC Health Serv Res. 2021 May 21;21:484. doi: 10.1186/s12913-021-06512-2 (PMC8138921; doi:10.1186/s12913-021-06512-2)
Supplement: Supplementary file 3 — Additional file 3: Table 3. Correlation between latent factors in Hawassa comprehensive Specialized Hospital, Hawwasa, Ethiopia, 2019. This shows if whether the five latent factors correlate with each other or not, which shows there is a high similarity in the items if there is a higher correlation. [file 12913_2021_6512_MOESM3_ESM.docx]

# Validity and reliability of the Amharic version of supportive care needs survey - short form 34 among cancer patients in Ethiopia

Tsion Afework^*^, Abigiya Wondimagegnehu , Natnael Alemayehu , Eva Johanna Kantelhardt^,^ Adamu Addissie

Table 3: Correlation between latent factors in Hawassa comprehensive Specialized Hospital, Hawwasa, Ethiopia, 2019.

| **Factor** |  | **Factor** | **Correlation** |
| --- | --- | --- | --- |
| PCS ^a^ | <--> | SEX ^e^ | 0.133 |
| PDI ^b^ | <--> | SEX | 0.059 |
| HSI ^c^ | <--> | SEX | 0.19 |
| PSY ^d^ | <--> | SEX | 0.303 |
| PDI | <--> | PCS | 0.369 |
| HSI | <--> | PCS | 0.813 |
| PSY | <--> | PCS | 0.615 |
| HIS | <--> | PDI | 0.242 |
| PSY | <--> | PDI | 0.355 |
| PSY | <--> | HIS | 0.6 |

^a^ patient care and support, ^b^ physical and daily living, ^c^ Health information, ^d^ Psychological, ^e^ sexuality
