# Supplementary material for: Validity and reliability of the Amharic version of supportive care needs survey - short form 34 among cancer patients in Ethiopia
Source: BMC Health Serv Res. 2021 May 21;21:484. doi: 10.1186/s12913-021-06512-2 (PMC8138921; doi:10.1186/s12913-021-06512-2)
Supplement: Supplementary file 4 — Additional file 4: Table 4. Reliability of the scale of SCNS-26 tool in Hawassa comprehensive Specialized Hospital, Hawassa, Ethiopia, 2019. Reliability was checked for only the remaining 26 items, which the result shows, all the construct had good reliability, which yields the same result on repeated measurements. [file 12913_2021_6512_MOESM4_ESM.docx]

# Validity and reliability of the Amharic version of supportive care needs survey - short form 34 among cancer patients in Ethiopia

Tsion Afework^*^, Abigiya Wondimagegnehu , Natnael Alemayehu , Eva Johanna Kantelhardt^,^ Adamu Addissie

Table 4: Reliability of the scale of SCNS-26 tool in Hawassa comprehensive Specialized Hospital, Hawassa, Ethiopia, 2019

| **Construct** | **No of Items** | **Cronbach’s alpha** |
| --- | --- | --- |
| Overall reliability test for the validated items | 26 items | 0.932 |
| Psychologic domain | 10 items | 0.994 |
| Health system & information domain | 5 items | 0.854 |
| Physical and daily living domain | 5 items | 0.887 |
| Patient care and support domain | 3 items | 0.799 |
| Sexuality domain | 3 items | 0.914 |
